# Albumin platelet product as a novel score for liver fibrosis stage and prognosis

**DOI:** 10.1038/s41598-021-84719-3

**Published:** 2021-03-05

**Authors:** Koji Fujita, Kazumi Yamasaki, Asahiro Morishita, Tingting Shi, Joji Tani, Noriko Nishiyama, Hideki Kobara, Takashi Himoto, Hiroshi Yatsuhashi, Tsutomu Masaki

**Affiliations:** 1 Department of Gastroenterology and Neurology, Faculty of Medicine, Kagawa University, 1750-1 Ikenobe Miki Kita, Kagawa, 761-0793 Japan; 2 Clinical Research Center, National Hospital Organization Nagasaki Medical Center, Omura, Japan; 3 Department of Medical Technology, Kagawa Prefectural University of Health Sciences, Takamatsu, Japan

**Keywords:** Risk factors, Laboratory techniques and procedures

## Abstract

Fibrosis-4 index, a conventional biomarker for liver fibrosis stage, is confounded by age and hepatitis activity grade. The current retrospective multicenter study aimed to formulate the novel indices of liver fibrosis by mathematically combining items of peripheral blood examination and to evaluate ability of prognosis prediction. After a novel index was established in a training cohort, the index was tested in a validation cohort. Briefly, a total of 426 patients were enrolled in a training cohort. Albumin and platelet most strongly correlated to fibrosis stage among blood examination. Albumin platelet product (APP) = Albumin × platelet/1000 could differentiate the four stages of liver fibrosis (*p* < 0.05). APP indicated fibrosis stage independent from hepatitis activity grade. A cut-off value = 4.349 diagnosed cirrhosis with area under ROC more than 0.8. Multivariate analysis revealed that smaller APP independently contributed to HCC prevalence and overall mortality. The results were validated in another 707 patients with HCV infection. In conclusion, APP was not confounded by age or hepatitis activity grade contrary to Fibrosis-4 index. APP is as simple as physicians can calculate it by pen calculation. The product serves physicians in managing patients with chronic liver disease.

## Introduction

Liver disease brings the world approximately 2 million of annual deaths^1^. One half of the liver disease-related deaths attributes to cirrhosis, the most progressed status of liver fibrosis. Evaluation of liver fibrosis stage enables a physician to predict and prevent patients from several complications of cirrhosis including esophagogastric varix, ascites, and hepatic encephalopathy expected in the future^2^. Hepatocellular carcinoma (HCC), the sixth leading malignancy and the third common cause of cancer death^3^, also typically complicates patients with cirrhosis^4^.

Liver biopsy examination used to be the gold standard for staging liver fibrosis.

However, noninvasive strategies to estimate fibrosis stage have already been replacing it^5^. The most inexpensive and simple modality should be several indices combining some items from complete blood count and liver function test, as represented by Fibrosis-4 index (FIB-4)^6,7^.

However, FIB-4 index has been reported to be confounded by age and hepatitis activity grade^8,9^. Furthermore, the index is difficult to calculate without ready-made online calculators. In the current retrospective multicenter study, we formulated a novel index of the liver fibrosis stage and prognosis by mathematically combining two items of peripheral blood examination in a training cohort, and validated their clinical significance in a validation cohort.

## Results

### Characteristics of the training cohort

According to diagram in Supplementary Fig. S1 online, a total of 426 patients comprising 252 of HCV patients, 27 of HBV patients, 52 of PBC patients, and 95 of AIH patients were enrolled in this study (Table 1). Based on the liver biopsy examinations, 128 patients had pointed out stage 1 fibrosis; 149 had stage 2 fibrosis; 114 had stage 3 fibrosis and 35 had stage 4 fibrosis. Among them, 336 patients were followed up for 1 year or more. The longest follow up period was 32 years. Hepatocellular carcinoma (HCC) was pointed out in 45 patients and 42 ones died.

**Table 1:** Baseline characteristics and follow up information of a training cohort.

Fibrosis stage	Total	F0–1	F2	F3	F4	R squared	*P* value
Patient number	426	128	149	114	35	−	−
Age	55 (44–64)	55 (46–64)	54 (42–62)	55 (44–64)	63 (53–67)	0.0169	0.0071
Male/female	182/244	36/92	74/75	59/55	13/22	−	−
HCV/HBV/PBC/AIH	252/27/52/95	58/8/23/39	93/10/15/31	72/9/12/21	29/0/2/4	−	−
Platelet count (× 10^9^/l)	179 (136–221)	215 (174–252)	183 (145–226)	150 (115–194)	106 (74–162)	0.1553	< 0.0001
Total protein (g/l)	74 (69–79)	74 (70–79)	74 (70–80)	74 (69–80)	73 (67–77)	0.0035	0.2285
Albumin (g/l)	38 (34–42)	40 (35–42)	40 (37–42)	37 (34–40)	31 (27–37)	0.1406	< 0.0001
AST (U/l)	59 (37–98)	44 (29–75)	54 (38–96)	82 (51–120)	74 (50–107)	0.0001	0.8335
ALT (U/l)	76 (41–128)	49 (28–113)	76 (43–128)	101 (66–151)	70 (45–104)	0.0029	0.2706
Total bilirubin (µmol/l)	13.8 (10.3–18.9)	12.0 (9.02–15.5)	12.0 (10.3–17.1)	17.2 (12.0–22.2)	18.9 (12.0–29.1)	0.0503	< 0.0001
γGTP (U/l)	58 (29–129)	63 (26–126)	48 (23–107)	67 (45–151)	40 (22–81)	0.0022	0.3406
**Followed up cohort (at least 1 year)**							
Patient number	336	95	117	100	24	−	−
Follow up period (years)	10 (5–18)	9 (4–18)	11 (7–21)	10 (5–18)	8 (3–10)	−	−
HCC prevalence (Patient number, %)	45 (13.4)	5 (5.3)	14 (12.0)	16 (16.0)	10 (41.7)	−	−
Death (Patient number, %)	42 (12.5)	6 (6.3)	18 (15.4)	6 (6)	12 (50)	−	−

*AIH* autoimmune hepatitis, *HBV* hepatitis B virus, *HCC* Hepatocellular carcinoma, *HCV* hepatitis C virus, *PBC* primary biliary cholangitis.

### Generation of Albumin platelet product

To determine items of an equation, linear trend through fibrosis staging was evaluated for age, platelet, total protein, albumin, aspartate aminotransferase (AST), alanine aminotransferase (ALT), total bilirubin (T-Bil), and gamma-glutamyl transpeptidase (γGTP). Among them, age and T-Bil linearly increased, and platelet and albumin decreased (Table 1). R squares were greater than 0.1 in platelet and Alb. Age and T-Bil had lower R squares than 0.1. Combining T-Bil, platelet and albumin mathematically, four equations were generated; Alb × Plt/1000, Albumin platelet product (APP); Alb/T-Bil, Albumin bilirubin quotient; 10 × T-Bil/platelet, Bilirubin platelet quotient; Alb × Plt/(T-Bil × 100), Three math.

### Diagnostic ability of novel indices for liver fibrosis staging in the training cohort

**Figure 1:**
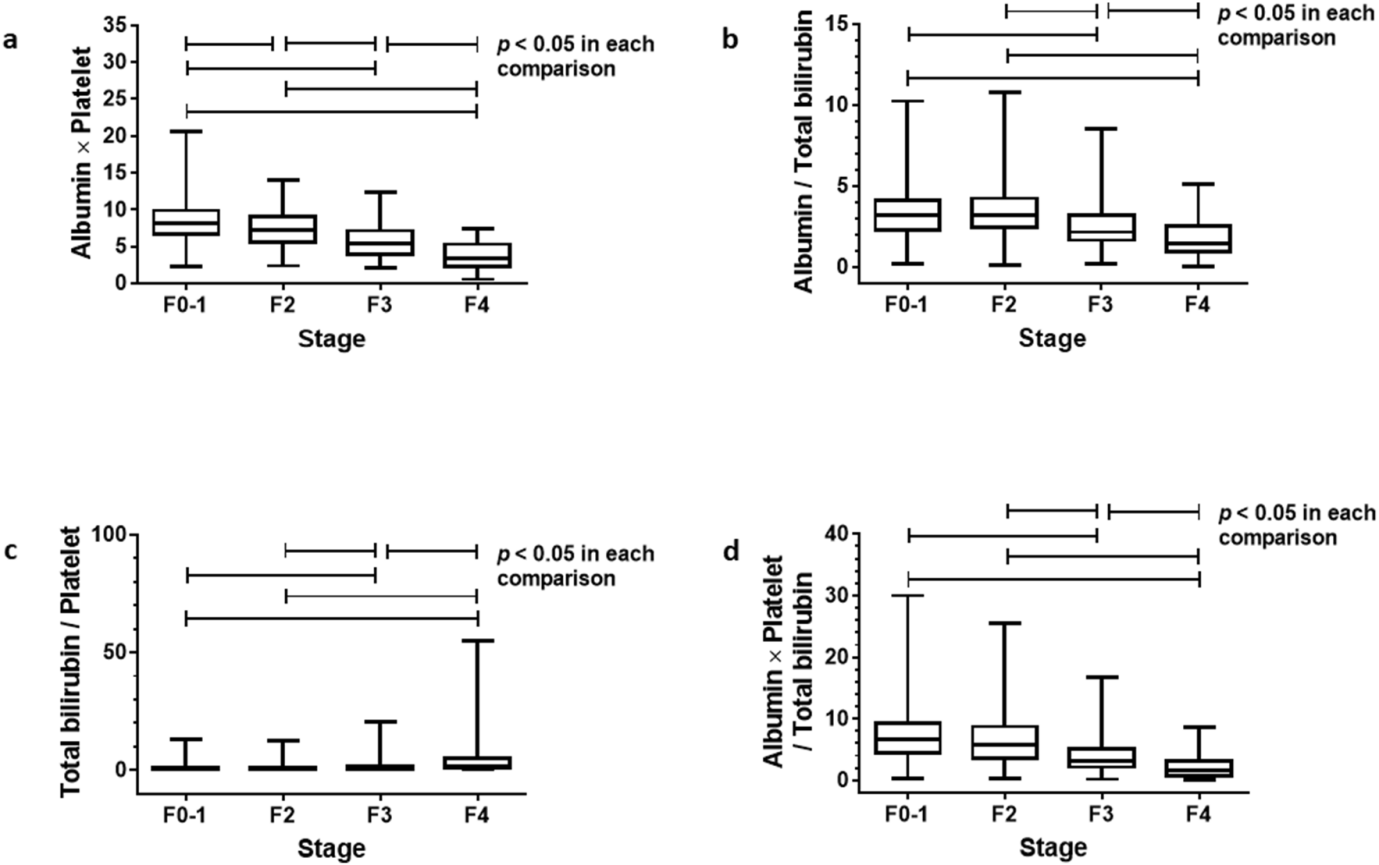
Newly generated fibrosis indices in each fibrosis stage in a training cohort. Median values of Albumin platelet product could differentiate each fibrosis stage (**a**). The other indices, Albumin bilirubin quotient (**b**), Bilirubin platelet quotient (**c**) and Three math; Albumin × platelet/(total bilirubin × 100) (**d**) distinguished stage 4 from stage 3 and stage 3 from stage 2, but failed to differentiate between stage 0–1 and 2 (*p* < 0.05). Data were analyzed using the Steel–Dwass test.

Differences in median values between two fibrosis stages were analyzed for four indices using Steel–Dwass test (Fig. 1). The results showed that the APP could differentiate any four stages (a). The albumin bilirubin quotient (b), bilirubin platelet quotient (c), and three math Alb × Plt/(T-Bil × 100) (d), could differentiate fibrosis stage 3 from stage 2 and stage 4 from stage 3, whereas they failed to differentiate between stage 1 and 2.

**Figure 2:**
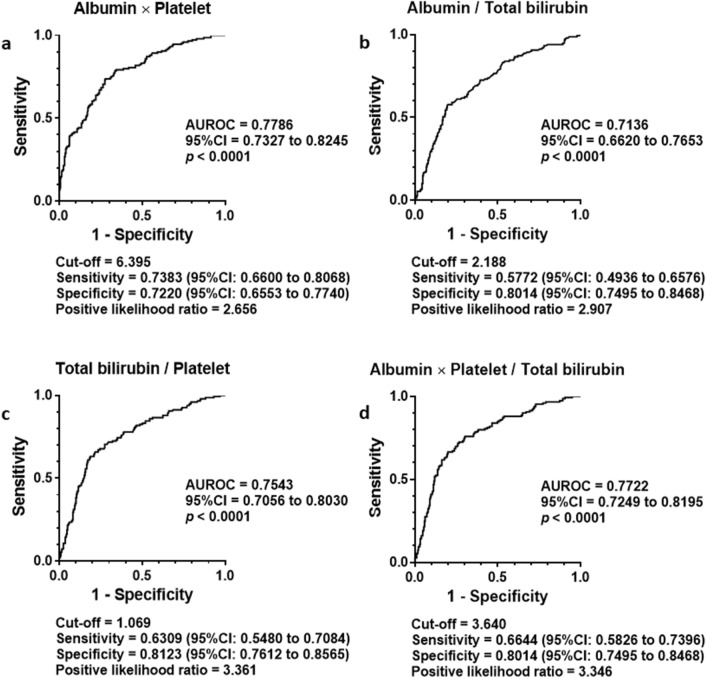
Differential diagnosis of advanced liver fibrosis by newly generated fibrosis indexes in the training cohort. ROC analysis to assess the ability of fibrosis indexes to differentiate advanced liver fibrosis (F3–4) from nonadvanced fibrosis (F1–2) yielded AUROC 0.7786 in Albumin platelet product (**a**), 0.7136 in Albumin bilirubin quotient (**b**), 0.7543 in Bilirubin platelet quotient (**c**) and 0.7722 in Three math; Albumin × platelet/(total bilirubin × 100) (**d**). A cut-off value of Albumin platelet product = 0.6395 presented 0.7383 of sensitivity and 0.7220 of specificity with 2.65 of positive likelihood ratio to differentiate advanced fibrosis from non advanced fibrosis (**a**). *P* values less than 0.05 were considered statistically significant.

Receiver operating characteristic (ROC) analysis was performed to assess the ability to distinguish advanced fibrosis (F3–4) from nonadvanced fibrosis (F0–2) and cirrhosis (F4) from noncirrhotic stages (F0–3). As shown in Fig. 2, area under ROC (AUROC) of four indices to distinguish advanced fibrosis from nonadvanced fibrosis was greater than 0.7 (a–d). A cut-off value of APP to differentiate F3–4 from F0–2 was determined at 6.395 with 0.7383 of sensitivity, 0.7220 of specificity, and 2.656 of positive likelihood ratio (a).

**Figure 3:**
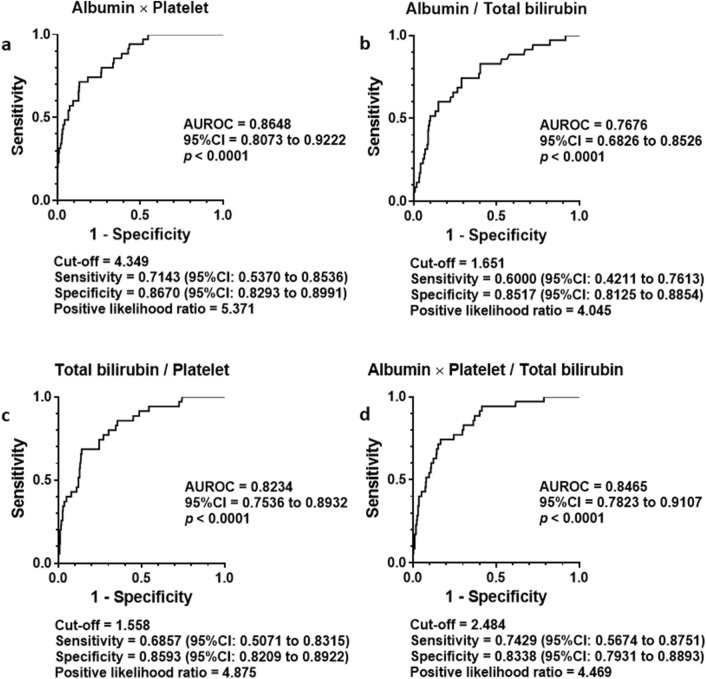
Differential diagnosis of liver cirrhosis by newly generated fibrosis indexes in the training cohort. ROC analysis revealed cirrhosis was differentially diagnosed from noncirrhotic status by Albumin platelet product (**a**), Albumin bilirubin quotient (**b**), Bilirubin platelet quotient (**c**) and Three math; Albumin × platelet/(total bilirubin × 100) (**d**). The greatest AUROC was presented by Albumin platelet product among them. Albumin platelet product = 4.349 determined by Youden Index presented 0.7143 of sensitivity and 0.8670 of specificity with 5.371 (**a**). *P* values less than 0.05 were considered statistically significant.

The AUROC of the indices to detect cirrhosis differentially from noncirrhotic stages resulted greater than 0.8 in APP (a), Bilirubin platelet quotient (c) and Three math (d) as shown in Fig. 3. The AUROC of Albumin bilirubin quotient stayed smaller than the others (b). A cut-off value of APP to differentiate F4 from F0–1 was determined at 4.349 with 0.7143 of sensitivity, 0.8670 of specificity, and 5.371 of positive likelihood ratio.

The greatest AUROC was presented by APP among four indices. The second was Three math. Based on the analyses above, APP and Three math were extracted as candidates of liver fibrosis staging.

### Comparison between APP and fibrosis-4 index in the training cohort

ROC analysis revealed, as shown in Supplementary Fig. S2 online, that AUROC of FIB-4 (a) to distinguish advanced fibrosis from nonadvanced fibrosis ranged between 0.7 and 0.8. The AUROCs to detect cirrhosis differentially from noncirrhotic stages were determined between 0.8 and 0.9 (b). Compared to FIB-4, APP revealed competitive in staging of liver fibrosis based on ROC analyses, as shown in Figs. 2a and 3a.

### Influence of hepatitis activity on fibrosis indices

Indices of liver fibrosis have been reported to fluctuate according to hepatitis activity grading^9^. Influence of hepatitis activity on Albumin platelet product was evaluated based on HCV-specific patients in the training cohort. As shown in Supplementary Table S1 online, 252 patients presented a distribution of liver fibrosis stage as follows; 58 of stage 1, 93 of stage 2, 72 of stage 3 and 29 of stage 4. Similar to results in the training cohort, Total bilirubin increased along with fibrosis progression while platelet and albumin decreased.

In 93 patients with stage 2, APP in grade 0–1 patients did not differ from that in grade 2 patients (Supplementary Fig. S2c online). In case of 72 patients with stage 3, APP was not significantly different between grade 1–2 and 3 patients (d). However, FIB-4 was significantly fluctuated in stage 2 and 3 patients (e, f).

### Prognosis prediction by APP in the training cohort

**Figure 4:**
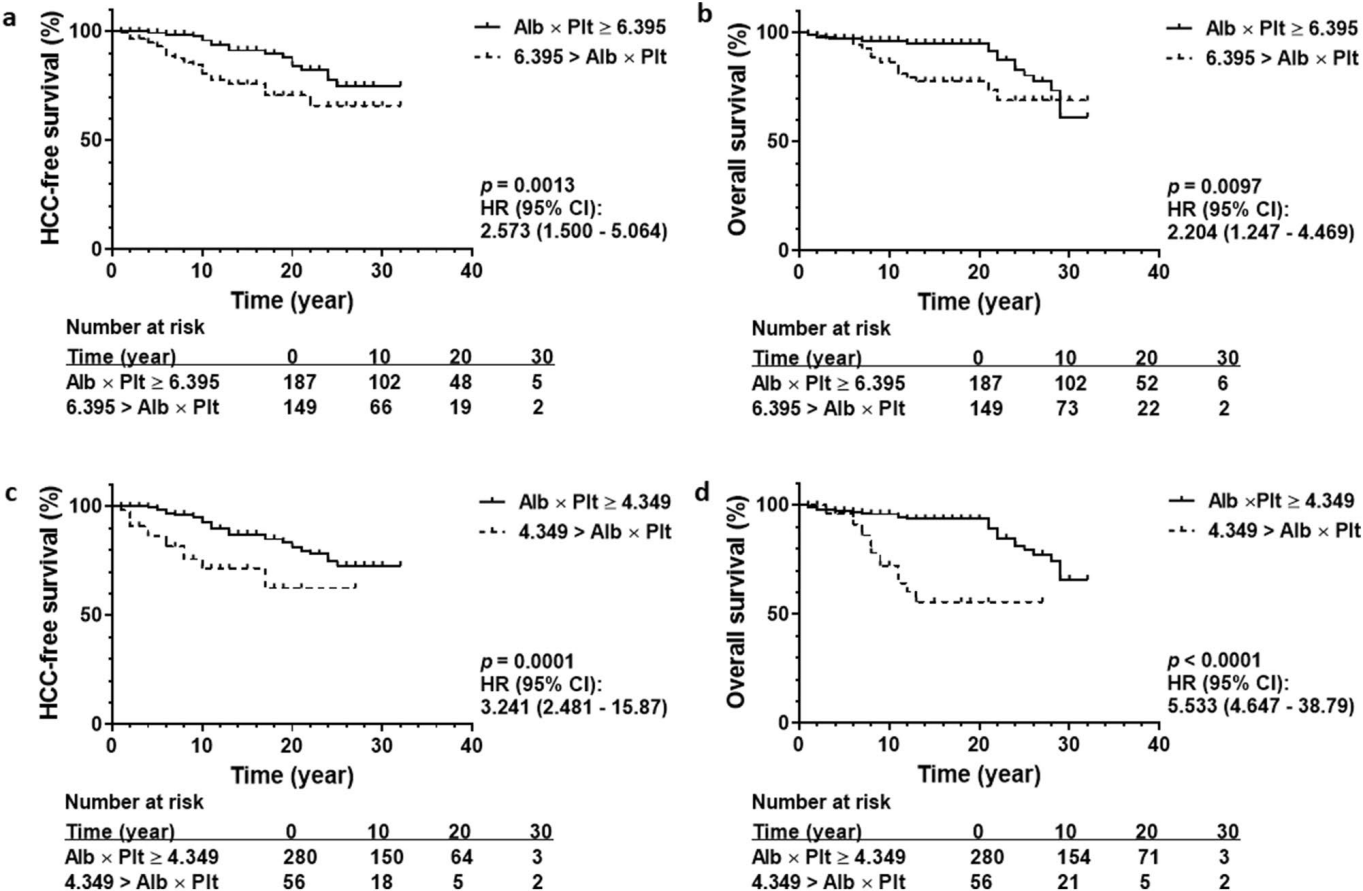
HCC-free survival and overall survival in a training cohort. Among 336 patients followed up for at least 1 year, 45 patients were complicated with HCC and 42 patients died. An Albumin platelet product cut-off value = 6.395 could differentiate HCC-free survival (**a**) and overall survival (**b**) in the Kaplan–Meier analysis. Albumin platelet product = 4.349 also predicted a difference in HCC-free survival (**c**) and overall survival (**d**) with statistical significance. *P* values less than 0.05 were considered statistically significant.

In total, 336 patients were followed up for at least one year in the training cohort. Kaplan-Meyer analysis was performed in the training cohort to estimate the contribution of the APP to HCC-free survival and overall survival. As shown in Fig. 4, a cut-off value = 6.395 could significantly differentiate HCC-free survival (a) and overall survival (b). Survival rates at 15 year were 91.2% and 75.9% for HCC free survival; 95.2% and 77.7% for overall survival. Another cut-off value = 4.349 also stratified HCC prevalence (c) and mortality (d) in the training cohort. Survival rates at 15 year were 87.1% and 71.5% for HCC free survival; 93.6% and 55.6% for overall survival.

Post-hoc power analysis resulted in a power = 0.975 for HCC-free survival and 0.998 for overall survival using a cut-off value = 6.395. When the training cohort was stratified using an alternative cut-off value = 4.349, HCC-free and overall survival was proved with power 0.808 and 1.000.

### A multivariate analysis in a training cohort

To investigate predictive ability of APP, Cox proportional hazard model was applied on follow up data of the training cohort. Concerning four variables, age, gender, etiology and APP, hazard ratios were analyzed to determine whether APP independently contribute to HCC prevalence and mortality in the training cohort. As shown in Table 2, both of APP < 6.395 and 4.349 significantly increased HCC prevalence and mortality. The proportional hazard model analysis was validated with 4 variables for 45 patients with HCC or 42 overall deaths (Table 1)^10^.

**Table 2:** Prediction of HCC prevalence and mortality by Albumin platelet product in a training cohort.

	HR	95% CI	*P* value
**Alb × Plt = 6.395 for HCC complication**			
Age	1.059	1.024–1.094	0.0008
Male/female	0.925	0.492–1.739	0.8090
Alb × Plt < 6.395/≧ 6.395	1.945	1.043–3.626	0.0363
AIH/HCV	0.167	0.039− 0.720	0.0165
HBV/HCV	1.968	0.649–5.969	0.2316
PBC/HCV	0.088	0.012–0.673	0.0191
**Alb × Plt = 6.395 for mortality**			
Age	1.077	1.035–1.120	0.0002
Male/female	1.007	0.475–2.135	0.9857
Alb × Plt < 6.395/≧ 6.395	2.020	1.021–3.997	0.0434
AIH/HCV	0.501	0.163–1.541	0.2282
HBV/HCV	< 0.001	−	0.9965
PBC/HCV	1.708	0.733–3.997	0.2148
**Alb × Plt = 4.349 for HCC complication**			
Age	1.059	1.025–1.094	0.0007
Male/female	1.062	0.559–2.020	0.8542
Alb × Plt < 4.349/≧ 4.349	2.961	1.503–5.834	0.0017
AIH/HCV	0.154	0.036–0.664	0.0121
HBV/HCV	2.225	0.746–6.639	0.1515
PBC/HCV	0.090	0.012–0.678	0.0194
**Alb × Plt = 4.349 for mortality**			
Age	1.074	1.032–1.118	0.0004
Male/female	1.283	0.604–2.723	0.5165
Alb × Plt < 4.349/≧ 4.349	4.063	1.984–8.324	0.0001
AIH/HCV	0.534	0.175–1.626	0.2692
HBV/HCV	< 0.001	−	0.9969
PBC/HCV	1.971	0.870–4.467	0.1041

*AIH* autoimmune hepatitis, *Alb* albumin, *Plt* platelet, *HBV* hepatitis B virus, *HCC* Hepatocellular carcinoma, *HCV* hepatitis C virus, *HR* hazard ratio, *PBC* primary biliary cholangitis, *95% CI* 95% confidence interval.

### Diagnostic ability of liver fibrosis staging in the validation cohort

To evaluate the diagnostic ability of liver fibrosis staging, the APP was calculated for each fibrosis stage in the validation cohort. As shown in Supplementary Fig. S3 online, the APP was able to differentiate the four stages of fibrosis (*p* < 0.05).

**Figure 5:**
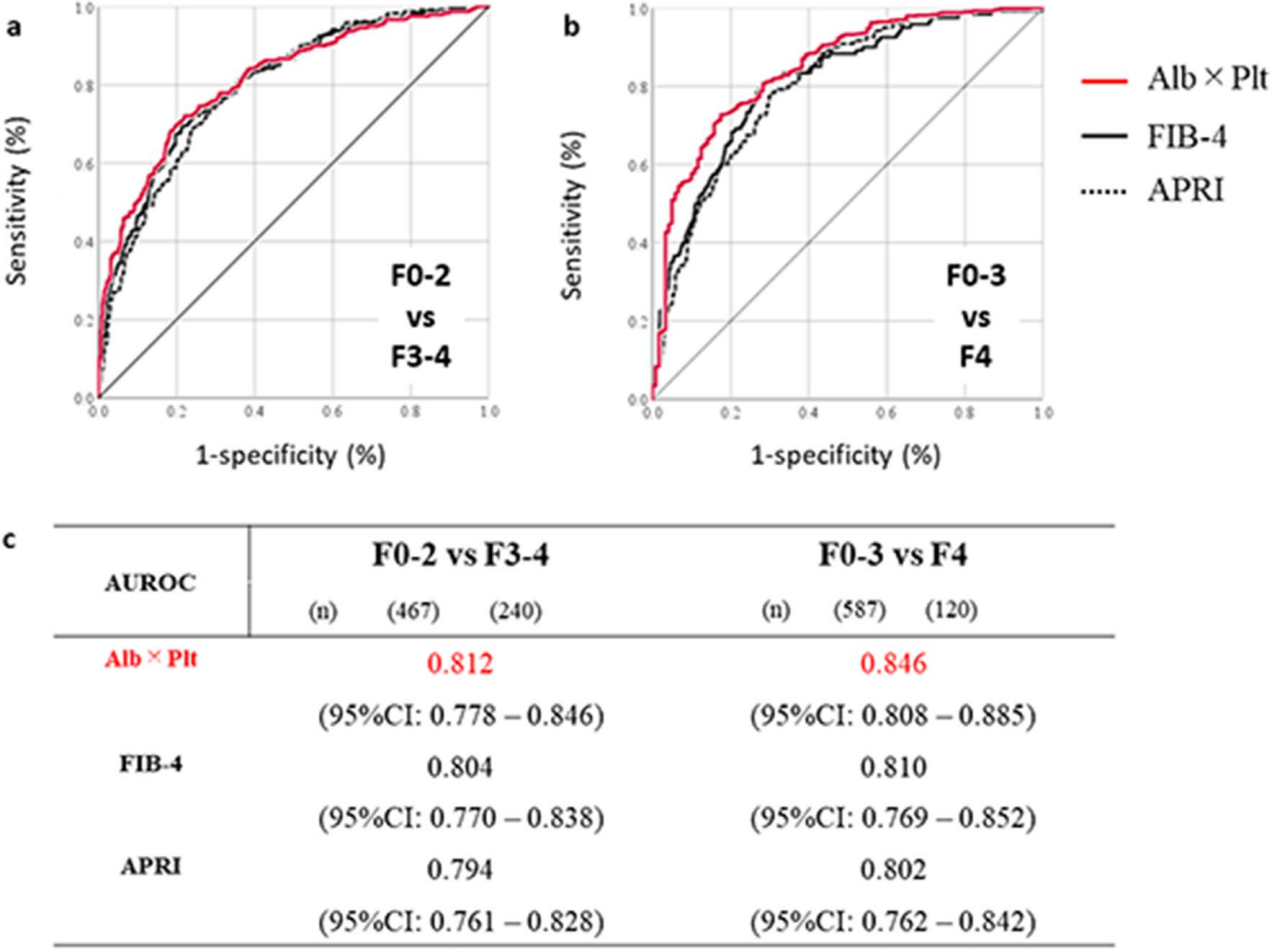
Differential diagnosis of liver fibrosis by the Albumin platelet product in a validation cohort*.* ROC analysis was performed to evaluate diagnostic abilities of Albumin platelet product for advanced liver fibrosis (F3–4) from nonadvanced fibrosis (F0–2) (**a**); for cirrhosis from noncirrhotic status (F0–3) (**b**). Albumin bilirubin product yielded the largest area under curve among three indices; Albumin bilirubin product, Fibrosis-4 index and APRI (**c**).

ROC analysis revealed that the AUROCs of the APP for distinguishing advanced fibrosis from nonadvanced fibrosis (a) and cirrhosis from noncirrhotic status (b) were greater than 0.8 (c), as shown in Fig. 5. The AUROCs of the APP were greater than that of FIB-4 or APRI (c). The diagnostic abilities of the APP with two cut-off values are summarized in Supplementary Table S2 online. Both cut-off values, APP = 6.395 and = 4.349, were characterized by negative predictive values relatively greater than 80%.

### Prognosis prediction by the APP in a validation cohort

The clinical impact of the APP on HCC-free survival and overall survival was confirmed using Kaplan–Meier analysis in the validation cohort through 15 years observation (Supplementary Fig. S4 online). Patient number of HCC complication and overall death at 15 year was 143 and 73. Each cut-off value, APP = 6.395 and 4.349, could differentiate HCC-free survival in 707 patients with HCV infection (a, b). Overall survival was also stratified by two cut-off values (c, d).

Post-hoc power analysis resulted in a power = 1.000 for four comparison above between greater and smaller APP groups.

The Cox proportional hazard model was also applied on the validation cohort using a stepwise method, as shown in Table 3. The performances of interferon therapy, sex, age, serum AFP and WFA^+^-M2BP levels were included in the multivariate analyses. The results showed that APP < 6.395 contributed to a greater risk of HCC complication. APP < 4.349 also indicated increased prevalence of HCC and overall death. Number of variables did not exceed 10 times of HCC patients or overall death^10,11^.

**Table 3:** Prediction of HCC prevalence and mortality by Albumin platelet product in a validation cohort.

		Hazard ratio (95% CI)	*P* value
**Alb × Plt = 6.395 for HCC complication**			
Alb × Plt	< 6.395/≧ 6.395	1.87 (1.25–2.79)	0.002
IFN therapy	Non-SVR/none	0.72 (0.50–11.02)	0.063
	SVR/none	0.13 (0.05–0.29)	< 0.001
Sex	Male/female	2.11 (1.50–2.97)	< 0.001
Age	≧ 57/< 57	1.90 (1.28–2.83)	0.001
AFP	≧ 7/< 7 ng/mL	2.10 (1.38–3.18)	0.001
WFA^+^-M2BP	≧ 2.86/< 2.86	2.22 (1.51–3.27)	< 0.001
**Alb × Plt = 6.395 for mortality**			
Alb × Plt	< 6.395/≧ 6.395	1.68 (0.95–2.98)	0.076
IFN therapy	Non-SVR/none	0.55 (0.34–0.91)	0.021
	SVR/none	0.18 (0.06–0.51)	0.001
Sex	Male/female	1.74 (1.09–2.79)	0.021
Age	≧ 57/< 57	2.93 (1.56–5.50)	0.001
AFP	≧ 7/< 7 ng/mL	1.77 (0.98–3.21)	0.058
WFA^+^-M2BP	≧ 2.86/< 2.86	2.75 (1.42–4.23)	0.001
**Alb × Plt = 4.349 for HCC complication**			
Alb × Plt	< 4.349/≧ 4.349	1.64 (1.10–2.45)	0.015
IFN therapy	Non-SVR/none	0.75 (0.52–1.07)	0.114
	SVR/none	0.13 (0.06–0.31)	< 0.001
Sex	Male/female	2.12 (1.51–2.99)	< 0.001
Age	≧ 57/< 57	2.06 (1.39–3.06)	< 0.001
AFP	≧ 7/< 7 ng/mL	2.31 (1.53–3.50)	< 0.001
WFA^+^-M2BP	≧ 2.86/< 2.86	2.20 (1.48–3.27)	< 0.001
**Alb × Plt = 4.349 for mortality**			
Alb × Plt	< 4.349/≧ 4.349	1.81 (1.06–3.11)	0.030
IFN therapy	Non-SVR/none	0.58 (0.35–0.97)	0.037
	SVR/none	0.19 (0.07–0.54)	0.002
Sex	Male/female	1.77 (1.10–2.84)	0.018
Age	≧ 57/< 57	3.14 (1.67–5.88)	< 0.001
AFP	≧ 7/< 7 ng/mL	1.89 (1.05–3.40)	0.035
WFA^+^-M2BP	≧ 2.86/< 2.86	2.29 (1.30–4.02)	0.004

*HCC* hepatocellular carcinoma, *IFN* interferon, *SVR* sustained viral response, *95% CI* 95% confidence interval, *WFA*^+^*-M2BP* Wisteria *floribunda* agglutinin-positive Mac-2 binding protein.

## Discussion

The current multicenter study presented that (1) APP is able to diagnose fibrosis stage, especially, advanced liver fibrosis and cirrhosis without confounding by age or hepatitis activity grade. (2) Smaller APP independently correlates with greater HCC prevalence and mortality.

Including age in its equation, FIB-4 is reported to overestimate fibrosis stage in senior patients^12^. Thus, a cut-off value of FIB-4 is proposed for each age group^8^. However, APP is not confounded by age because the index is not based on age.

Fibrosis staging by APP equals in accuracy to FIB-4. Diagnostic ability of APP was lined by stability of it against hepatitis activity grading. We previously reported that FIB-4, APRI, enhanced liver fibrosis score (ELF score) and Wisteria *floribunda* agglutinin-positive Mac-2 binding protein (WFA^+^-M2BP) fluctuated in an identical fibrosis stage according to activity grade^9^. APP might be more suitable for fibrosis staging than other indices.

FIB-4 consists of four items, age, AST, platelet, and square root of ALT. Calculating FIB-4 using an electronic calculator is not easy in general practice because the square root of ALT locates as one of Denominators in the equation. Therefore, customized calculators for FIB-4 have been open in the internet. The equation of APRI is simpler compared to FIB-4. However, diagnostic abilities of APRI for liver fibrosis were not competitive to that of FIB-4 in the current cohort.

In summary, the data probed that APP was as reliable as FIB-4 in liver fibrosis staging. In addition, the equation of APP is as simple as APRI because APP is calculated by two items alone. Physicians in office can calculate APP by an electronic calculator or even by pen calculation.

The limitations of the current study might lie in the fact that (1) because the current observation focused on patients with HCV infection patients, eligibility of APP in patients with other etiologies should be evaluated in further studies. (2) Although the influence of antiviral therapy on patients with HCV was evaluated in terms of HCC-free survival and overall survival, the prognostic impacts of nucleos(t)ide analogues for patients with HBV, ursodeoxycholic acid for patients with PBC, and steroidal agents for patients with AIH were not considered in the analyses. (3) In the training cohort, the baseline year at which liver biopsy was performed varied among some decades. Thus, the baseline year was quite variable, probably confounding the prognosis of patients in the training cohort. The validation cohort may have a much smaller risk of such confounding. (4) Finally, a prospective study is necessary to exclude any other potential biases originating from the retrospective nature of this study.

In conclusion, APP indicates liver fibrosis stage and prognosis in Japanese patients with chronic liver diseases, predominantly with HCV infection. The diagnostic accuracy of APP to differentiate fibrosis stage was competitive to that of FIB-4, and free from confounding by age or hepatitis activity. Furthermore, smaller APP independently contributes to HCC prevalence and mortality. APP enables physicians to manage patients with chronic liver diseases.

## Methods

### Study design

The current retrospective study investigated novel indices for liver fibrosis and prognosis consisting of two or three blood exams. Clinical information and pathological stage of liver fibrosis were available to readers of the novel index, but not available to the liver pathologists. Baseline complete blood count, biochemical test, and coagulation function test were performed within a week before performing the liver biopsy.

After a novel index was established in a training cohort, the index was tested in a validation cohort. Diagnostic potential for liver fibrosis stage was described by area under ROC curve, sensitivity, specificity and positive likelihood ratio. Prognosis prediction by a novel index was presented using two endpoints, prevalence of HCC and overall deaths. Multivariate analysis was performed to process potential confounders within allowable number of parameters ruled by number of events^10^. To control any potential biases in a training cohort, clinical significance of a novel index was evaluated in a validation cohort. Sample size was validated based on post-hoc power analysis. The study was performed according to STARD and STROBE statement^13,14^.

### Ethics

This study was conducted in accordance with the ethical principles of the Declaration of Helsinki and was approved by the Institutional Review Board at Kagawa University, Faculty of Medicine (Heisei-30-151)^15^. Informed consent was obtained from all subjects or, if subjects are under 18, from a parent and/or legal guardian.

### Generation of novel fibrosis indices

To select candidate blood exam items for a novel index, linear trend through fibrosis stage was evaluated for them. Considering R squares, two or three items were mathematically combined as a simple product or ratio.

### A training cohort

Japanese patients with HCV and HBV infection, primary biliary cirrhosis definite and probable autoimmune hepatitis patients who underwent percutaneous liver biopsy examinations for a clinical condition between 1986 and 2019 in Kagawa University Hospital, were consecutively enrolled. Patients who had hepatocellular carcinoma when the liver biopsy examinations were performed were excluded.

### Clinical data

The following clinical data were extracted from the participants’ medical records: age, gender, platelet count, AST, ALT, γGTP, T-Bil, total protein and albumin in blood examinations. T-Bil (mg/dl) was converted to T-Bil (µmol/l) according to the equation: T-Bil (mg/dl) × 17.1. FIB-4, a conventional liver fibrosis index, was calculated using the following equation: age × AST (U/l)/(Plt (10^9^/l) × √ALT (U/l))^16^. APRI was calculated using the following equation: 100 × (AST (U/l)/upper limit of normal AST values (U/l))/(Plt (10^9^/l)^17^.

HCV infection was confirmed by polymerase chain reaction, combined reverse transcription-PCR, or branched chain DNA probe assay. HBV infection was determined by positive HBsAg, HBeAg, HBeAb, HBcAb, or DNA polymerase described in medical records. Serological diagnosis of PBC was performed using an anti-mitochondrial antibody and an anti-mitochondrial M2 antibody^18^. Data for the anti-centromere antibody was also extracted for patients who were followed up for one year or more^19^. Definite and probable AIH was diagnosed according to IAIHG criteria revised in 1999^20^.

### Histopathological analysis

For HCV, HBV, and AIH samples of liver biopsies, the extent of fibrosis was assessed using a modified METAVIR score (modified from^21^) as follows: stage 1, portal or central fibrosis; stage 2, some septa; stage 3, many septa; stage 4, cirrhosis. The METAVIR grading system was used to assess hepatic inflammatory activity^22^. Pathological stage of PBC was evaluated using the Scheuer classification (stage 1, florid duct lesion; stage 2, ductular proliferation; stage 3, scarring; and stage 4, cirrhosis) by experienced pathologists who specialized in liver pathology^23,24^. Staging and grading were performed by experienced pathologists who specialized in liver pathology.

### A validation cohort

A validation cohort was identical from patients in a past report investigated for WFA^+^-M2BP, a serum biomarker of liver fibrosis^11^. As shown in the past report, the validation cohort comprised 707 patients with HCV infection, including 274 patients with fibrosis stage 0–1; 193 with stage 2; 120 with stage 3; and 120 patients with stage 4. All other clinical data of the validation cohort were identical to those in the past report.

### Statistical analysis

Continuous variables were presented as median and interquartile ranges. Mann–Whitney *U* test was used for comparison between average and median values. Kruskal‐Wallis' analysis of variance (ANOVA), followed by the Steel–Dwass post hoc test, was used to assess significant differences in terms of fibrosis stages (F0–1, F2, F3, and F4). Categorical variables were analyzed using Fisher’s exact test. Cut-off values in ROC analysis were determined using Youden index^25^. *P* < 0.05 was considered statistically significant.

For the training cohort, statistical analyses above were performed using GraphPad Prism 6 (GraphPad Software, La Jolla, CA) and EZR (Saitama Medical Center, Jichi Medical University, Saitama, Japan), a graphical user interface for R software (The R Foundation for Statistical Computing, Vienna, Austria)^26,27^, at Kagawa University.

For the validation cohort, the abovementioned statistical analyses were performed using SPSS statistical software version 26.0 (SPSS, Inc., Chicago, IL), JMP 14 (SAS Institute Inc., Cary, NC), and EZR at Nagasaki Medical Center.

## Supplementary Information

Supplementary Information 1.

Supplementary Figure S1.

Supplementary Figure S2.

Supplementary Figure S3.

Supplementary Figure S4.

## Abbreviations

AIH: Autoimmune hepatitis
APRI: AST to platelet ratio index
ELF score: Enhanced liver fibrosis score
FIB-4: Fibrosis-4 index
HCC: Hepatocellular carcinoma
HBV: Hepatitis B virus
HCV: Hepatitis C virus
PBC: Primary biliary cholangitis
WFA^+^-M2BP: Wisteria *floribunda* agglutinin-positive Mac-2 binding protein
