# Supplementary material for: Albumin platelet product as a novel score for liver fibrosis stage and prognosis
Source: Sci Rep. 2021 Mar 5;11:5345. doi: 10.1038/s41598-021-84719-3 (PMC7935926; doi:10.1038/s41598-021-84719-3)
Supplement: Supplementary file 1 — Supplementary Information 1. [file 41598_2021_84719_MOESM1_ESM.docx]

**Title:** Albumin platelet product as a novel score for liver fibrosis stage and prognosis.

**Running title:** Albumin platelet product

**Authors:** Koji Fujita^1,^*, Kazumi Yamasaki^2^, Asahiro Morishita^1^, Tingting Shi^1^, Joji Tani^1^, Noriko Nishiyama^1^, Hideki Kobara^1^, Takashi Himoto^3^, Hiroshi Yatsuhashi^2^, Tsutomu Masaki^1^

**Affiliation:** ^1^Department of Gastroenterology and Neurology, Faculty of Medicine, Kagawa University; ^2^Clinical Research Center National Hospital Organization Nagasaki Medical Center; ^3^Department of Medical Technology, Kagawa Prefectural University of Health Sciences

***Corresponding author:** Koji Fujita

**Address:** 1750-1 Ikenobe Miki Kita Kagawa 761-0793 Japan

**E-mail:** 92m7v9@med.kagawa-u.ac.jp

**Tel.:** +81-87-891-2156

**Fax:** +81-87-891-2158

**The total number of figures and tables:** 9 figures, 5 tables

**Key words:** Biomarkers; Cirrhosis; Hepatitis C, Chronic; Hepatocellular carcinoma; Mortality

**Supplementary Legends**

**Supplementary Figure S1 online. Diagram of training cohort**

**Supplementary Figure S2 online.** **Comparison between Albumin platelet product and *Fibrosis-4 index* in a training cohort**

ROC analysis confirmed the ability of Fibrosis-4 index to differentiate advanced liver fibrosis (F3-4) (a) and cirrhosis (b) in a training cohort. The area under curves were smaller compared to that of Albumin platelet product, as shown in Fig. 2a and 3a.

Influence of hepatitis activity on Albumin platelet product was evaluated based on HCV-specific patients presented in Supplementary Table S1. Albumin platelet product in fibrosis stage 2 was not statistically different between activity grade 0-1 group and grade 2 group (**c**). In stage 3, Albumin platelet product for grade 3 group did not differ from that of grade 1-2 group (**d**). In case of Fibrosis-4 index, the median values were significantly different depending on activity grade in stage 2 (**e**) and stage (**f**). Data were analyzed using Mann-Whitney-*U* test. *P* values less than 0.05 were considered statistically significant.

**Supplementary Figure S3 online. Fibrosis staging by the Albumin platelet product in a validation cohort**

The Albumin platelet product could significantly differentiate stages 0-1, 2, 3, and 4. Data were analyzed using the Steel-Dwass test. *P* values less than 0.05 were considered statistically significant.

**Supplementary Figure S4 online. HCC-free survival and overall survival of patients with HCV infection in a validation cohort**

A validation cohort was divided by a cut-off value = 6.395 and = 4.349. HCC-free survival was significantly differentiated by Albumin platelet product = 6.395 (**a**) and = 4.349 (**b**). Two cut off values also stratified overall survival with statistical significance (**c**, **d**). *P* values less than 0.05 were considered statistically significant.

**Supplementary Tables**

**Table S1 online. Baseline characteristics of HCV patients in a training cohort**

| Fibrosis stage | Total | F0-1 | F2 | F3 | F4 | R squared | *P* value |
| --- | --- | --- | --- | --- | --- | --- | --- |
| Patient number | 252 | 58 | 93 | 72 | 29 | - | - |
| Age | 54 | 52 | 52 | 57 | 63 | 0.0700 | < 0.0001 |
|  | (43 - 62) | (38 - 60) | (42 - 60) | (47 - 63) | (55 - 66) |  |  |
| Male/Female | 149/103 | 29/29 | 58/35 | 49/23 | 13/16 | - | - |
| Platelet count (× 10^9^/L) | 168 | 190 | 179 | 138 | 94 | 0.2610 | < 0.0001 |
|  | (125 - 202) | (173 - 230) | (139 - 211) | (106 - 174) | (63 - 133) |  |  |
| Total protein (g/L) | 74 | 73 | 75 | 73 | 74 | 0.0010 | 0.5980 |
|  | (70 - 78) | (70 - 77) | (71 - 79) | (69 - 77) | (67 - 79) |  |  |
| Albumin (g/l) | 39 | 41 | 41 | 37 | 34 | 0.2568 | < 0.0001 |
|  | (36 - 42) | (39 - 43) | (39 - 43) | (34 - 40) | (28 - 38) |  |  |
| AST (U/l) | 55 | 36 | 52 | 74 | 76 | 0.0459 | 0.0005 |
|  | (36 - 86) | (23 - 57) | (35 - 78) | (51 - 103) | (49 - 105) |  |  |
| ALT (U/l) | 77 | 4 | 76 | 101 | 70 | 0.0009 | 0.6200 |
|  | (42 - 127) | (25 - 93) | (42 - 128) | (71 - 146) | (41 - 100) |  |  |
| Total bilirubin (µmol/L) | 13.7 | 12 | 12.0 | 17.1 | 18.8 | 0.1341 | < 0.0001 |
|  | (10.3 - 18.8) | (8.6 - 15.4) | (10.3 - 15.5) | (12.0 - 20.5) | (12.8 - 29.1) |  |  |
| γGTP (U/l) | 44 | 31 | 38 | 65 | 40 | 0.0130 | 0.0660 |
|  | (24 - 86) | (19 - 67) | (22 - 79) | (39 - 119) | (22 - 77) |  |  |
| Activity grade (0-1/2/3) | (129/74/20) | (50/7/1) | (64/29/0) | (15/38/19) | - | - | - |

HCV, hepatitis C virus

**Table S2 online. Diagnostic abilities of Albumin platelet product for liver fibrosis in a validation cohort**

| Alb × Plt = 6.395 |  |  |
| --- | --- | --- |
| Stage | F0-2 (n=467) | F3-4 (n=240) |
| ≥ 6.395 (n) | 319 | 148 |
| < 6.395 (n) | 53 | 187 |
| Sensitivity(%) | 77.9 (72.1 – 83.0) | |
| Specificity(%) | 68.3 (63.9 – 72.5) | |
| Positive likelihood ratio | 2.46 (2.12 – 2.85) | |
| PPV(%) | 55.8 (50.3 – 61.2) | |
| NPV(%) | 85.8 (81.8 – 89.1) | |
| Alb × Plt = 4.349 |  |  |
| Stage | F0-3 (n=587) | F4 (n=120) |
| ≥ 4.349 (n) | 536 | 57 |
| < 4.349 (n) | 51 | 63 |
| Sensitivity (%) | 52.5 (43.2 – 61.7) | |
| Specificity (%) | 91.3 (88.7 – 93.5) | |
| Positive likelihood ratio | 6.04 (4.42 – 8.26) | |
| PPV(%) | 55.3 (45.7 – 64.6) | |
| NPV(%) | 90.4 (87.7 – 92.6) | |

PPV, positive predictive value; NPV, negative predictive value
