# Supplementary figures and images for: Albumin platelet product as a novel score for liver fibrosis stage and prognosis
Source: Sci Rep. 2021 Mar 5;11:5345. doi: 10.1038/s41598-021-84719-3 (PMC7935926; doi:10.1038/s41598-021-84719-3)

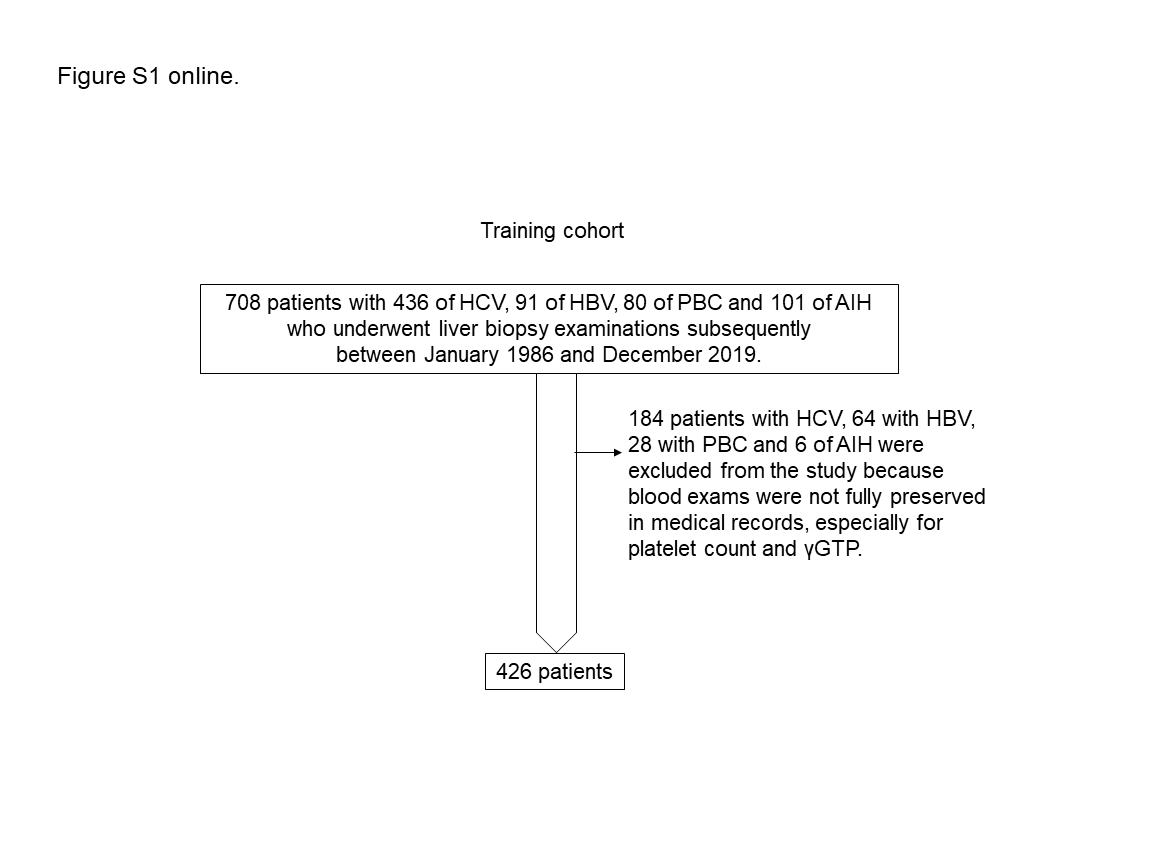

Supplement: Supplementary file 2 — Supplementary Figure S1. [file 41598_2021_84719_MOESM2_ESM.tif]

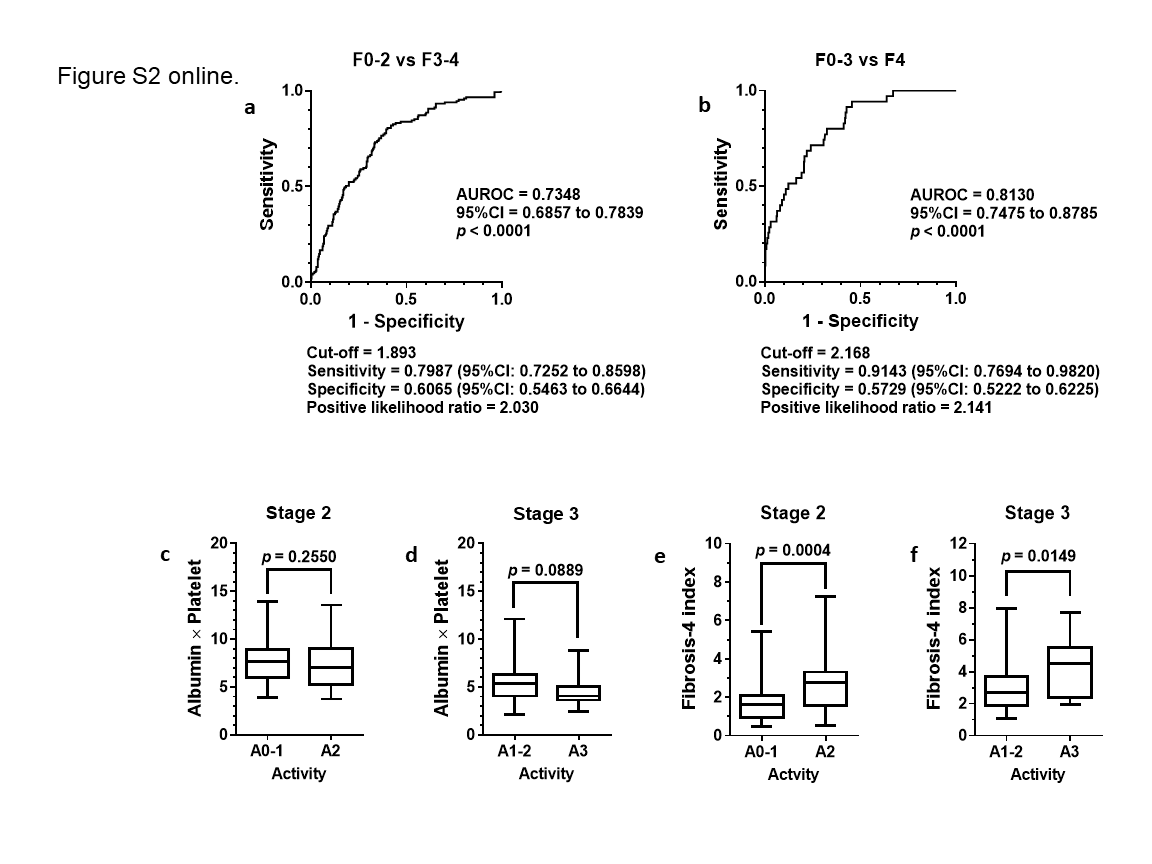

Supplement: Supplementary file 3 — Supplementary Figure S2. [file 41598_2021_84719_MOESM3_ESM.tif]

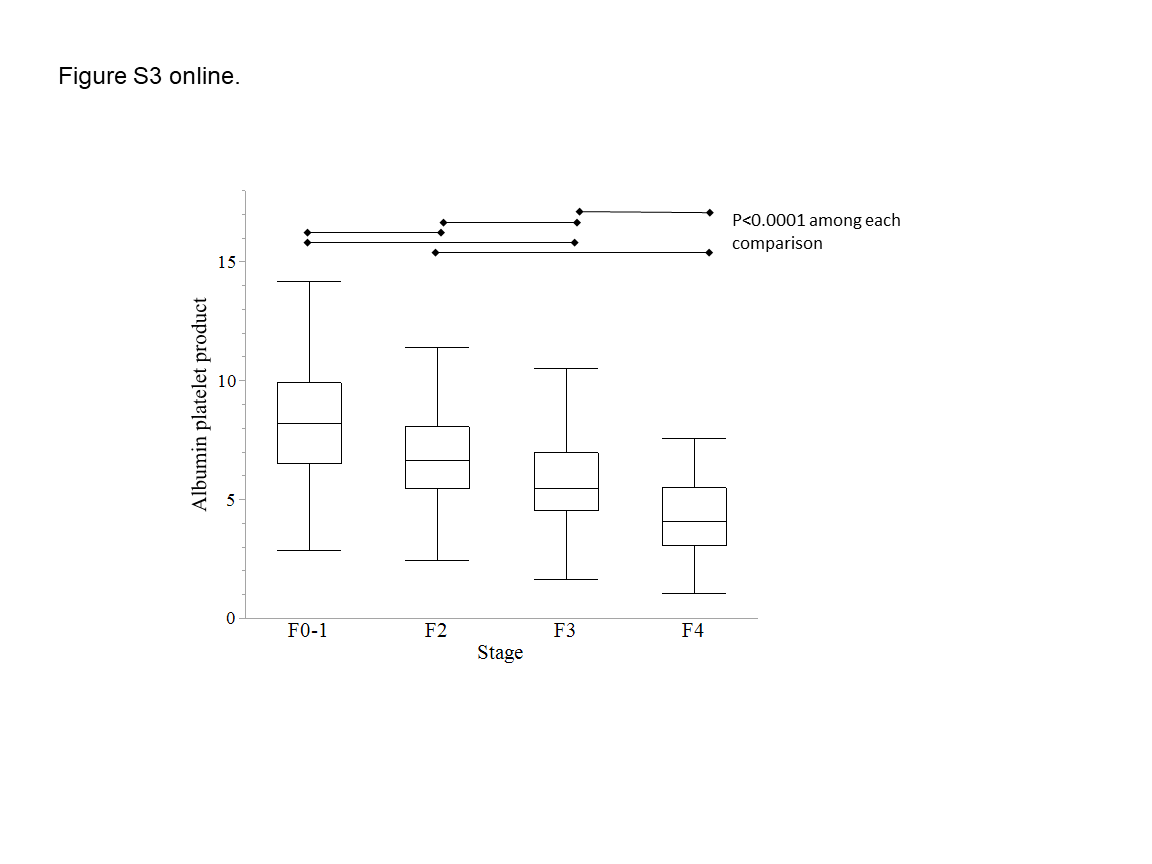

Supplement: Supplementary file 4 — Supplementary Figure S3. [file 41598_2021_84719_MOESM4_ESM.tif]

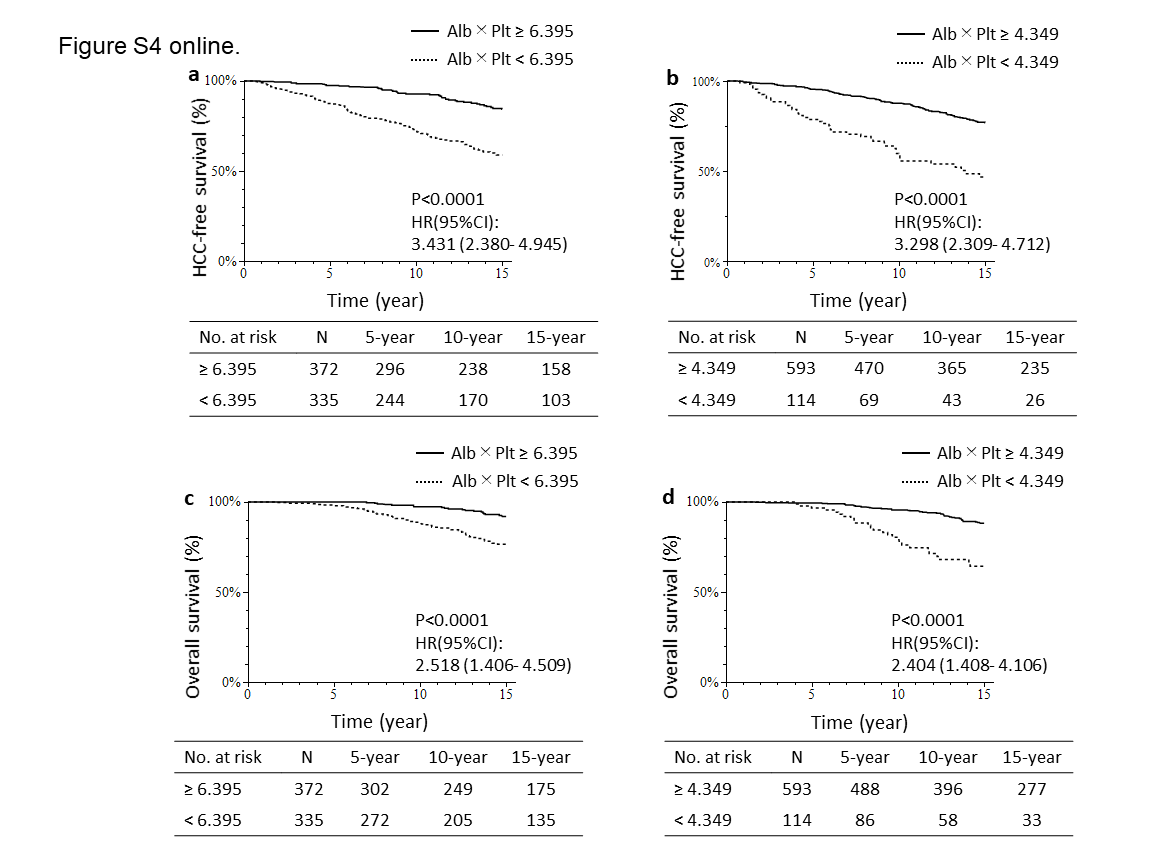

Supplement: Supplementary file 5 — Supplementary Figure S4. [file 41598_2021_84719_MOESM5_ESM.tif]
